# Supplementary material for: The Systems Biology Research Tool: evolvable open-source software
Source: BMC Syst Biol. 2008 Jun 29;2:55. doi: 10.1186/1752-0509-2-55 (PMC2446383; doi:10.1186/1752-0509-2-55)
Supplement: Additional file 1 — SBRT Archive. An archive of the current version of the Systems Biology Research Tool. [file 1752-0509-2-55-S1.zip › sbrt-1.4.0/doc/developers_guide/api/sbrt/shell/io/ComiFile.html]

ComiFile


|  |  |  |
| --- | --- | --- |
| **Overview** \| **Package** \| **Class** \| **Use** \| **Tree** \| **Deprecated** \| **Index** \| **Help** | |  |
| **PREV CLASS**   **NEXT CLASS** | **FRAMES**    **NO FRAMES**     **All Classes** |  |
| SUMMARY: NESTED \| FIELD \| CONSTR \| METHOD | DETAIL: FIELD \| CONSTR \| METHOD |  |


---


## sbrt.shell.io Class ComiFile<K,V>

```
java.lang.Object
  sbrt.shell.io.AbstractMapInputFile<K,V>
      sbrt.shell.io.ComiFile<K,V>
```

**Type Parameters:**: `K` - the key type.: `V` - the value type.

**All Implemented Interfaces:**: java.lang.Iterable<java.util.Map<K,V>>, InputFile<java.util.Map<K,V>>, MapInputFile<K,V>, MapTextFile<K,V>, MapTextInputFile<K,V>, SbrtFile

---

``` public class ComiFile<K,V> extends AbstractMapInputFile<K,V> ```

This class is used to read column-oriented map input files.

**Author:**
:   This class was written and documented by
    Jeremiah Wright while in the Wagner lab.

---

**Constructor Summary**

`ComiFile(java.lang.String fileName,
FileFormat fileFormat,
MapFormat<K,V> mapFormat)`
  
          Constructs a new row-oriented map input file from the
provided arguments.

`ComiFile(java.lang.String fileName,
FileFormat fileFormat,
java.util.Set<? extends K> requiredKeys,
MapFormat<K,V> mapFormat)`
  
          Constructs a new row-oriented map input file from the
provided arguments.

`ComiFile(java.lang.String fileName,
MapFormat<K,V> mapFormat)`
  
          Constructs a new row-oriented map input file from the
provided arguments.


| **Method Summary** | |
| --- | --- |
| `java.util.Set<K>` | `getKeys()`             Returns the map keys (column headers) contained in this file. |
| `static SparseDoubleMatrix<java.lang.Integer,java.lang.String>` | `getMatrix(ComiFile<java.lang.String,java.lang.Double> file)`             Reads the provided column-oriented map input file and returns its contents as a sparse matrix. |
| `java.util.Iterator<java.util.Map<K,V>>` | `iterator()`             Returns an iterator over this file. |
| `java.util.Iterator<java.util.Map<K,V>>` | `iterator(int mapIndex)`             Returns an iterator over this file beginning at the specified index. |

**Methods inherited from class sbrt.shell.io.AbstractMapInputFile**

`close, getCanonicalName, getMapFormat, getOriginalName, getRequiredKeys, isClosed, lineIterator`

**Methods inherited from class java.lang.Object**

`clone, equals, finalize, getClass, hashCode, notify, notifyAll, toString, wait, wait, wait`

**Constructor Detail**

### ComiFile

```
public ComiFile(java.lang.String fileName,
                FileFormat fileFormat,
                java.util.Set<? extends K> requiredKeys,
                MapFormat<K,V> mapFormat)
```

:   Constructs a new row-oriented map input file from the
    provided arguments.

    **Parameters:**: `fileName` - the name of the file to read.: `fileFormat` - the format of the file.: `requiredKeys` - the keys that are required to be in the file.: `mapFormat` - the format used to parse the file.

---


### ComiFile

```
public ComiFile(java.lang.String fileName,
                FileFormat fileFormat,
                MapFormat<K,V> mapFormat)
```

:   Constructs a new row-oriented map input file from the
    provided arguments.

    **Parameters:**: `fileName` - the name of the file to read.: `fileFormat` - the format of the file.: `mapFormat` - the format used to parse the file.

---


### ComiFile

```
public ComiFile(java.lang.String fileName,
                MapFormat<K,V> mapFormat)
```

:   Constructs a new row-oriented map input file from the
    provided arguments. The file format is assumed to be
    `FileFormat.TEXT`.

    **Parameters:**: `fileName` - the name of the file to read.: `mapFormat` - the format used to parse the file.


**Method Detail**

### getMatrix

```
public static SparseDoubleMatrix<java.lang.Integer,java.lang.String> getMatrix(ComiFile<java.lang.String,java.lang.Double> file)
```

:   Reads the provided column-oriented map input file and returns its
    contents as a sparse matrix. The row indices are numbered 1 to n, and
    column indices are the map keys.

    :   **Parameters:**: `file` - the file to be read. **Returns:**: the contents of the provided file as a matrix.

---


### getKeys

```
public java.util.Set<K> getKeys()
```

:   Returns the map keys (column headers) contained in this file.

    :   **Returns:**: the map keys (column headers) contained in this file.

---


### iterator

```
public java.util.Iterator<java.util.Map<K,V>> iterator()
```

:   Returns an iterator over this file.

    :   **Returns:**: an iterator over this file.

---


### iterator

```
public java.util.Iterator<java.util.Map<K,V>> iterator(int mapIndex)
```

:   Returns an iterator over this file beginning at the specified
    index. Indices begin at `0`, and commented or blank lines are
    ignored.

    :   **Parameters:**: `mapIndex` - the index of the map at which this iterator begins. **Returns:**: an iterator over this file beginning at the specified index. **Throws:**: `java.lang.IndexOutOfBoundsException` - if the provided index exceeds the number of maps contained in the file.


---


|  |  |  |
| --- | --- | --- |
| **Overview** \| **Package** \| **Class** \| **Use** \| **Tree** \| **Deprecated** \| **Index** \| **Help** | |  |
| **PREV CLASS**   **NEXT CLASS** | **FRAMES**    **NO FRAMES**     **All Classes** |  |
| SUMMARY: NESTED \| FIELD \| CONSTR \| METHOD | DETAIL: FIELD \| CONSTR \| METHOD |  |


---
